# The g-C_3_N_4_@CdO/ZnO ternary composite: photocatalysis, thermodynamics and acute toxicity studies

**DOI:** 10.1016/j.heliyon.2022.e11612

**Published:** 2022-11-17

**Authors:** Sintayehu Berhanu, Haftom Gebremariam, Samuel Chufamo

**Affiliations:** a Department of Chemistry, Bonga University, P.O. Box 334, Bonga, Ethiopia; b Department of Biology, Bonga University, P.O. Box 334, Bonga, Ethiopia; c Department of Chemistry, Wolaita Sodo University, P.O. Box 138, Wolaita Sodo, Ethiopia

**Keywords:** G-C_3_N_4_@ZnO/CdO, Kinetics, Photocatalyst, Thermodynamics, Toxicity

## Abstract

Binary and ternary nanocomposites (NCs) were synthesized by precipitation and through facile one-pot ultrasonic assisted methods to serve as photocatalysts for treatment of wastewater as well as their toxicity toward aquatic organism (Nile tilapia). The crystalline structure, band gap energy and functional groups of these materials were characterized by XRD, UV-Vis, and FT-IR instrumental techniques. Based on the UV-Vis study, the band gap of ZnO/CdO (ZC) to hybrid g-C_3_N_4_@ZnO/CdO (GZC) nanocomposite was reduced from 3.41 eV to 3.21 eV, suggesting good charge carrier mobility. Photocatalytic degradation performances of ZC and GZC were further assessed by conducting methyl red (MR) photodegradation reaction using UV light. The highest degradation efficiency was achieved for GZC NCs (97.78%) than ZC (89.41%) in 2 h. The values of free energy, and enthalpy were negative; showing spontaneous photodegradation of MR. The kinetics of photodegradation follows pseudo-first-order reaction with rate order of 0.0713 min^−1^. The HO∗ and O_2_∗ were main active species for the photodegradation of MR. The toxicity of NCs calculated and the lethal concentration (LC_50_) was 113 ppm after 12 h.

## 1 Introduction

The fast increment of organic pollutants from paper, rubber and textile industry unfavorably influence the quality of water, environmentally unfriendly and harsh to aquatic life. Furthermore, some dyes are either toxic or carcinogenic to human being and aquatic organism. Owing to their harmfulness and slow degradation they are categorized as environmentally hazardous materials [1, 2, 3].

Methyl red (MR) is widely used organic dye in the textile, industrial paints, plastics and cosmetics industry. A large amount of this is lost during the dyeing process and is released in the effluent water streams from the above industries. Because of its aromatic structure, MR has physico-chemical, thermal and optical stability and it’s difficult in biodegradation process. Therefore, decolorization and detoxification of organic dye effluents have taken an increasingly important environmental significance in recent years [3, 4, 5].

Photocatalysis is one of the most chemical routes for eradication of environmental toxins. Heterogeneous semiconductor metallic oxide photocatalysts play important roles in many industrial and innovative processes including environmental and biomedical applications [2, 3, 4, 5, 6]. Among numerous metal oxides, ZnO has excellent photocatalysis. When ZnO catalysts are exposed to UV-Vis light with photons of energy equal or greater than their band gap energy the generated electron-hole pairs can induce the formation of reactive oxygen species which directly participates in the oxidative processes leading to the degradation of organic pollutants [4, 5, 6, 7].

A photocatalytic degradation of toxic compound by ZnO looks to be the most fascinating and has been a topic of research interest for degradation and decolorization of water pollutants to non-hazardous product. ZnO is broadly studied because of wide bandgap (3.37 eV), stability against photoirradiation, ease of doping, high conductivity, and chemical stability [7, 8, 9].

But, the photocatalytic activity of ZnO is restricted to poor kinetics due to very low electron mobility as well as shorter ion diffusion path possibly its low electronic conductivity allow in large charge transfer resistance [10]. It has been improved by various methods such as doping [7], coupling [8] or a combination of both [2].

Adding of metal or metallic oxide nanoparticles through doping advances charge separation, due to the presence of delocalized electrons. ZnO with metal ions such as Mn, Pb, Cd or Ag significantly increased the photocatalytic enhancement with the decreasing electron hole pair recombination rate [2, 3, 4]. From the different metallic oxide, CdO is appropriate candidate for photocatalytic uses. Cadmium oxide (CdO) is semi-conductor transparent material with a direct band gap of 2.2–2.5 eV and 1.36–1.98 eV, unique optical and optoelectrical properties.

Yousef et al. reported that the modification of ZnO with CdO give two band gap in photoluminescence spectra which have the best photocatalytic activity with a ratio of 39.4:60.6(CdO:ZnO) wt% [6]. The ZnO/CdO nanocomposite (NC) gives that the degradation of organic pollutant under visible light irradiation for 4.5 h was achieved due to the delay of back reaction between CdO and ZnO which produces large number of charge carriers which would increase the efficiency of degradation [11, 12].

Currently, many research group have established hybrid semiconductors as effective photocatalysts, including carbon nitride [13] and polyaniline [14]. From different n-type semiconducting polymers, graphitic carbon nitride (g-C_3_N_4_) is essential polymer with high electrical conductivity, ease of synthesis, narrow bandgap energy (∼2.7 eV), biocompatibility and extensional conjugated electronic structure. Furthermore, modifying ZnO/CdO with g-C_3_N_4_ can improve the photo-generated charge movement efficiency at the interface between the nanoparticles [14, 15, 16].

The current work is engaged with synthesis of a new photocatalyst for the degradation of MR dye from waste water. Initially, the g-C_3_N_4_@CdO/ZnO NC was synthesized then the morphology, crystalline sizes, functional group as well as the band gap energy were measured by XRD, FT-IR and UV-vis spectroscopic technique. The synthesized photocatalyst are then used for studying the decolorization and degradation of MR in aqueous solution. Bioassay tests are central for estimating the usefulness of the synthesized materials in waste water treatment. The exposure to the NC and their potential effects could be assessed in freshwater using Nile tilapia (*Oreochromis niloticus*). Finally, the acute toxicity, uptake and accumulation of different concentrations of NC is studied and discussed after exposure for 12 h, 24 h, 48 h and 72 h.

## 2 Experimental

### 2.1 Chemical

Zinc chloride (ZnCl_2_) (95%), cadmium chloride monohydrate (CdCl_2_·H_2_O) (99%) and urea were purchased from Sigma-Aldrich and used as received without any further purification. NaOH and HCl were of analytical grade which purchased from SISCO Research Laboratories Pvt. Ltd. Methyl red was purchased from BDH. All the suspensions and dye solutions were prepared with doubly distilled water (DW).

### 2.2 Synthesis of photocatalyst

#### 2.2.1 Synthesis of g-C_3_N_4_

A simple thermal condensation method was used to synthesis g-C_3_N_4_ [17]. 30 g of urea was weighed and placed in a porcelain crucible which covered with aluminum foil sheets and calcined at 550 °C for 3 h with a heating rate of 10 deg/min at atmospheric condition, when the temperature reduced to room temperature, a yellowish powder was obtained.

#### 2.2.2 Synthesis of ZnO/CdO

ZnO/CdO (ZC) NC was synthesized using methods reported elsewhere with little modification [18]. Initially, 2 M ZnCl_2_ (13 ml) solution was taken in a 250 ml round bottom flask, and added with 1 M CdCl_2_ (13 ml) solution. Then the mixture was kept under constant stirring, which allows the formation of colloidal suspension of (Cd(OH)_2_ with Zn(OH)_2_). Next, the temperature of reaction bath was gradually increased to 70–80 °C. Finally, 2 M NaOH solution was added slowly, up to the precipitate was completely formed. The precipitate was continuously stirred for 5 h. The residue was washed several times with doubly distilled water until the filtrate turn into neutral and dried in oven at 60 °C for 12 h.

#### 2.2.3 Synthesis of g-C_3_N_4_@ZnO/CdO

Initially, equal amounts of ZnO and CdO were dispersed together in distilled water using ultra-sonication for 2 h. Next, the powder was collected by filtration and washed several times with distilled water. The filtered powder was allowed to dry at 80 °C and denoted as ZnO/CdO. Then, the dispersion of g-C_3_N_4_ nanoparticles on the surface of the ZC was achieved by a facile room-temperature ultrasonic-assisted route. Typically, 0.1 g of g-C_3_N_4_ nanoparticle was dissolved in 100 mL distilled water, and then 1.0 g of binary ZC NC was added with this solution and ultra-sonicated for 3 h. Then, the NC was filtered, washed several times by distilled water and finally allowed to dry at 60 °C for 12 h, and the final product was denoted as g-C_3_N_4_@CdO/ZnO (GZC).

#### 2.2.4 Characterization of the photocatalysts

The FT-IR spectra of the photocatalyst were measured with a Thermo Scientific Nicolet iS50 FT-IR spectrometer in the range of 400–4000 cm^−1^. The Ultraviolet-visible (UV-vis) absorption spectra of the photocatalyst as well as the concentration of the dyes were investigated by the Sanyo UV-Vis spectrophotometer model (SP65, UK) spectrophotometer in the whole UV-Visible region (200–800 nm). XRD patterns were measured using a BRUKER D8 (West Germany and equipped with Cu Kα radiation λ = 1.5405 Å) in the scan range 2θ between 10 and 90° at room temperature.

#### 2.2.5 Photocatalytic activity

The catalytic activities of the as-synthesized CdO/ZnO and hybrid g-C_3_N_4_@CdO/ZnO photocatalysts were allowed to studies methyl red (MR) dye degradation under dark and UV light. For studying the methyl red degradation, 0.1 g/L of CdO/ZnO and hybrid g-C_3_N_4_@CdO/ZnO photocatalysts and 0.03 g/L of 250 mL aqueous solution of MR was taken in a reactor tube, separately. The mixture was stirred for 30 min in the dark environment to ensure adsorption/desorption equilibrium. Then the suspension was exposed to UV irradiation (15 W UV-A 223 lamp, Sylvania) with continuous stirring. The first and irradiated methylene red concentration of solutions was checked by UV–Vis spectrophotometer in 20 min time interval at the maximum wavelength of MR solution. Percent (%) of degradation was calculated using the following formula [17].

(1) $$\%\;of\;degradation=\frac{C_{o}-C_{t}}{C_{o}}\times 100$$

where, is $C_{0}$ is concentration of dye at initial stage and $C_{t}$ concentration of dye at time t.

#### 2.2.6 Factors affecting photocatalytic degradation of MR

The effect of photocatalyst load, CdO/ZnO and hybrid g-C_3_N_4_@CdO/ZnO was studied to find the finest amount of the catalyst from 0.01 to 1.0 g/L keeping other parameters such as initial dye concentration (0.4 g/L) and pH constant (pH 6.4). The effect of concentration of MR solution was investigated by changing the initial MR concentration also keeping pH = 6.4 and photocatalyst load 0.2 g/L. To obtain pH with higher degradation efficiency the effect of pH was studied by adjusting the pH of the initial MR solution from 2 to 12 with 1.0 M HCl and NaOH solutions in 100 mL of photocatalytic solution.

### 2.3 Kinetics and thermodynamics of photocatalysis

The kinetic analysis for order of the photocatalytic degradation process is calculated with graphical method. Mostly, it can be intended that organic pollutant photocatalytic degradation obey pseudo-first-order reaction with rate explained by

(2) $$r=-\frac{dC\left(t\right)}{dt}=kC\left(t\right)$$

At lower concentration the rate constant was calculated using Langmuir-Hinshelwood model [19].

(3) $$r=\ln\left(\frac{C_{t}}{C_{o}}\right)=-kt$$

where r - is rate of reaction, Ct - is the concentration at a given time, Co-is the concentration at initial time, t is the reaction time (min) and k-observed rate constant in min^−1^. Thermodynamics analysis of MR Photodegradation is also very useful to know the energetic contemplation of a reaction. Subsequently the temperature of reaction could affect the rate of degradation.

Therefore the effect of temperature on photocatalytic degradation was investigated at different temperatures in the range from 290 K to 310 K.

### 2.4 Acute toxicity assessment

The fate of GZC evaluated in the test medium as well as their relative acute toxicity in Nile tilapia (*Oreochromis niloticus*) fish species using OECD 203 test guidelines for supporting risk assessment of this nanocomposite [20]. The fishes were purchased from a local supplier and assessments were carried out in rebuilt fresh water that was aerated to maintain the dissolved oxygen level. Sample of GZC NCs were suspended in 25 mL of Nile tilapia fish media to prepare stock concentrations of 1.0 g/L. The solution was then suspended and dispersed via an ultrasonic bath then, concentrations of 0.01, 0.05, 0.1, 0.15, and 0.5 g/L of GZC NC were tested to determine their acute toxicity. Ten fish were randomly placed into the exposure containers then fish death rates were measured at intervals of 12, 24, 48, and 72 h. Dissolved oxygen (DO) (10.54 mg/l), pH (= 7.14) and Water temperature (24 °C) were measured daily through the experiment. The concentration–response plot of GZC NCs was used to evaluate to what amount the toxicity of the GZC NCs could be accounted.

## 3 Result and discussion

### 3.1 FT-IR analysis

**Figure 1:**
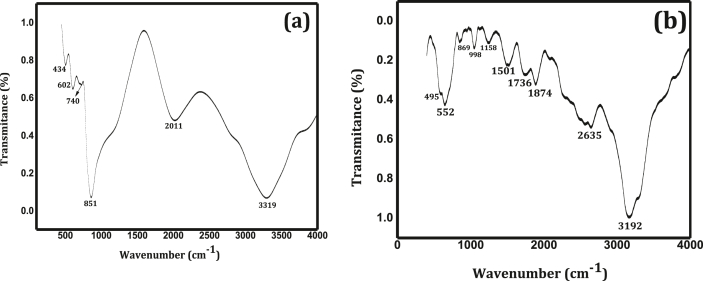
FT-IR spectra of: ZnO/CdO (a) and g-C_3_N_4_@ZnO/CdO (b).

FT-IR spectrum is used to identify molecular geometry, functional groups, and different interactions between molecular compounds. The FT-IR spectra of ZnO/CdO and g-C_3_N_4_@ZnO/CdO are carried out in the wavenumber range between 400 and 4000 cm^−1^ and shown in Figure 1a and b. The band appeared at 3319 cm^−1^ in Figure 1a and the weak deformation band at 2011 cm^−1^ are related to the symmetric stretching and bending vibration of H_2_O molecule. The main peak for Cd–O and Zn–O nanoparticles was appear in the region from 420 to 850 cm^−1^ [19,21]. The peak for ZnO is observed at 434 cm^−1^ in Figure 1a which confirms the transformation of ZnCl_2_ to hexagonal ZnO and the peak observed at 740 cm^−1^ is an indication of cubic CdO molecule vibrations [22]. The overtones of CdO and ZnO are observed at 851 cm^−1^ (Figure 1a) and 861 cm^−1^ (Figure 1b) which indicate that the formation of tetrahedral coordination of Zn and Cd nanoparticles [23, 22, 24].

The main peaks for stretching vibration of aromatic sp^2^ C=N and sp^3^ C=N (bending vibrations) of heptazine-derived repeating units of g-C_3_N_4_ given in the region between 900 and 1700 cm^−1^ (Figure 1b) [25, 26]. The absorption peak centered at 869 cm^−1^ and the broad peak at 3192 cm^−1^ were assigned as the deformation mode of N–H in amino groups and the stretching vibration of free N–H in the bridging C–NH–C units [27, 28]. The peak at 851 cm^−1^ of ZC, exhibits significant red shift in GZC at 552 cm^−1^, due to the quantum confinement effect [28].

### 3.2 XRD analysis

**Figure 2:**
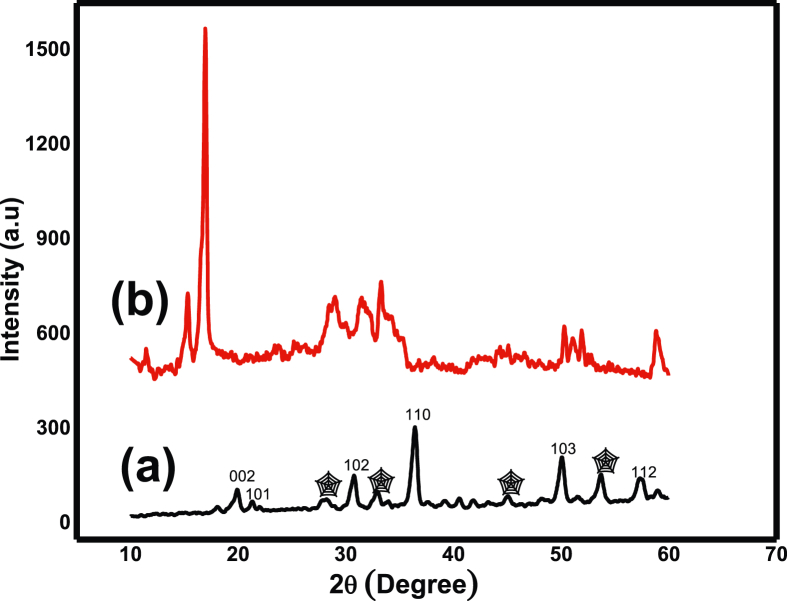
XRD pattern of ZnO/CdO (a) and g-C_3_N_4_@ZnO/CdO nanocomposite (b).

The crystallinity of the ZC and GZC hybrid composites were characterized using XRD. The diffraction pattern of ZC (Figure 2a) shown that the characteristic peaks of ZnO was appeared in the 2θ values of 20.56°, 22.27°, 30.76°, 36.40°, 49.76° and 57.26° related to (100), (002), (101), (102), (110) and (103) plane values. This related with hexagonal structure of ZnO NPs. The little new peaks connected with CdO which show the insertion of cadmium and the peak intensity also diminishes as compared to pure ZnO, this is due to stacking of CdO on the ZnO wurzite structure (Card No: 36-1451). For CdO four Bragg peaks were seen, the diffraction peak at 2θ = 28.02° (111), 32.81° (200), 45.01 (220) and 53.86 (222) related with cubic crystalline phase of CdO (DB card number of 1011003).

As compared to the ZC, two new peaks at 2θ = 18.52° and 2θ = 30.23° were found in GZC hybrid (Figure 2b), which can be assigned to the (100) and (002) plane of g-C_3_N_4_. The locations and shapes of ZC characteristic peaks of GZC are unaffected compared with those of pure ZC. It specifies that modification with g-C_3_N_4_ does not influence structure of ZC, which is fundamental for photocatalytic properties of as-prepared hybrid photocatalyst. Moreover, the average crystalline size was calculated by Scherrer’s equation (D=λ/2sinθ) [29]. In the presence of g-C_3_N_4_ nanoparticles, crystal size of nanocomposites decreased from 10.2 nm for ZC to 8.2 nm for GZC nanocomposites. This may due an interaction between the delocalized lone pair of electron in g-C_3_N_4_ to the inorganic filler [30, 31, 32].

### 3.3 UV-vis spectra analysis

**Figure 3:**
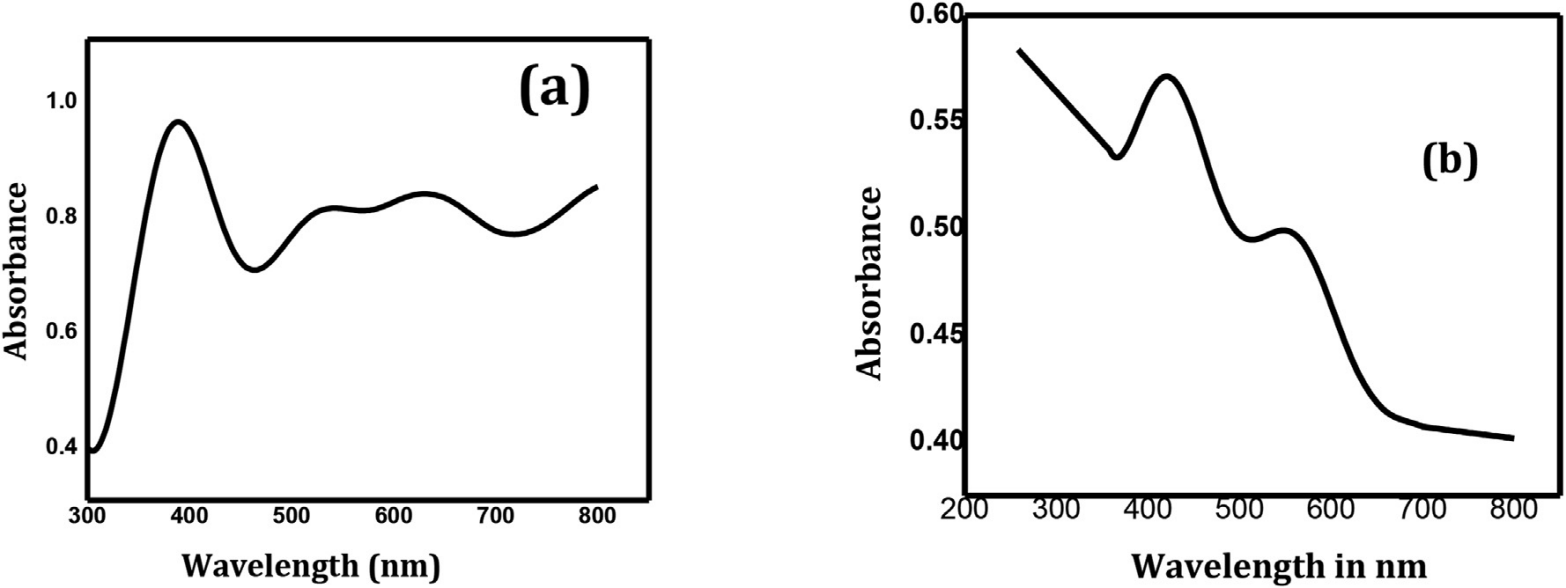
UV-Vis absorption spectra of ZC (a) and GZC (b).

The optical characteristics of the as-synthesized photocatalysts are characterized (Figure 3). Figure 3a and 3c demonstrates the absorption spectra of binary ZC and ternary GZC photocatalyst which have λmax at 401 and 502 nm. The band gaps were assessed using the Tauc plot which were given in Figure 3b and 3d.

The band gaps of ZC and GZC were calculated as 3.21 eV and 3.41 eV, respectively. The band gap energy of the ternary system decreases as relative to the binary ZC NC, this is variation of band gap arises due to the synergetic effect. GZC ternary NC is red shifted as compared to the ZC this is due to creation of localized energy levels by g-C_3_N_4_ in the band gap of ZC, with broadening of the band and strong UV-light absorption [33].

It is known that the photocatalytic activiy is based on the wavelength of light irradiation. Thus, the photocatalytic activity by GZC would be higher than ZC NCs with visible light irradiation [34].

### 3.4 Photocatalytic degradation of methyl red

#### 3.4.1 Comparison of photocatalytic activities of the nanocomposite

The photocatalytic activities of synthesized nanomaterials were tested under UV-light irradiation at the maximum absorption of λ = 425 nm. The adsorption/desorption equilibrium was done under dark for 30 min to use as blank and the percent degradation value of methyl red under visible irradiation was found after taking off the percent adsorption value without illumination. Generally, the photocatalytic activity of ternary NC (GZC) (97.78%) was found to be greater than the binary NC ZC (89.41%) under UV illumination.

**Figure 4:**
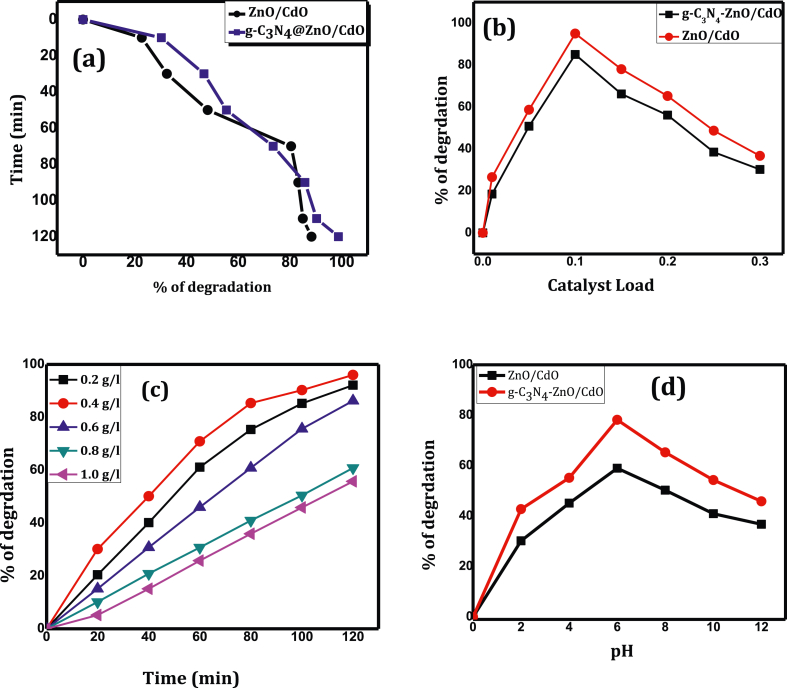
(a) Comparison of photocatalytic degradation of MR using ZnO/CdO (ZC) and g-C_3_N_4_@ZnO/CdO (GZC), (b) Effect of catalyst load (c) effect of initial concentration (d) Effect of pH of the solution on the degradation of methyl red (MR) dye.

This could be due to the possible retardation of the photochemical activity because some probable back reaction occurs between ZnO and CdO there by creating large number of charge carriers that would consequently increase the efficiency of degradation. Also, the GZC have more than two paths to form electron-hole pairs, this is due to the presence of three different interfaces, as well as the electron-hole recombination banned to the maximum in GZC. Hence, the enhanced photocatalysis of GZC might be due to effective loading of g-C_3_N_4_ on ZnO/CdO NC to generate UV sensitive hetero-junction which raises its photo absorption ability in the UV region. The course of MR degradation using the as-synthesized photocatalysts is given in Figure 4a.

#### 3.4.2 Effect of photocatalyst loading

The effect of photocatalyst load on the rate of degradation of MR was carried out by using different concentration of ZC and GZC NC in the range of 0.01–2.0 g/L. As shown in Figure 4b, degradation rate increases with increasing catalyst load from 0.01 to 0.1 g/L; further increases of catalyst amount from 0.15 g/L to 0.3 g/L however resulted in decreased degradation of MR. This occurs because the light intensity distribution may be non-uniform apparently subsequent from overloading. Therefore, the reaction rate would become lower. Also, at lower photocatalyst concentration, the degradation of the MR should be lower indicating that lesser transmitted radiation would only be used during photocatalysis [35]. The optimum catalyst concentration for ZC and GZC was found to be 0.12 and 0.11 g/L.

#### 3.4.3 Initial concentration of methyl red

The effect of the initial concentration of methyl red on its degradation was seen by taking different initial concentrations from 0.2 g/L to 1.0 g/L and setting other parameters constant (photocatalyst load 0.2 mg/L, pH = 6.5). The results are shown in Figure 4c. It can be observed from this figure that the % of degradation efficiency was increased with an increase in catalyst loading up to 0.4 g/L.

Additional increase in dye concentration decreased the % photo-degradation. The decrease in % of photo-degradation as the concentration increased can be due to the inadequate number of active sites exist when relative to the number of MR molecules exist at higher concentrations and shielding effect. This inhibits the interactions of photons of the light with the photodegradation system, thus ensuing to decreased photodegradation efficiency [36].

#### 3.4.4 Effect of pH

The pH is one of useful parameter of adsorption method it affects % of ionization of target pollutants, the surface charge of the adsorbent, and dissociation of functional groups on the active site of adsorbent [37]. To study the effect of pH on the degradation of MR, the pH of the solution was varied from 2 to 12 by adjusting with 1.0 M each of NaOH or HCl, by fixing other parameter constant (catalyst load 0.1 g/L and 0.4 g/L dye concentration), and the results are shown in Figure 4d.

The photodegradation results show that maximum adsorption and higher photocatalysis was gotten at pH 6.4. This is because the greater interaction among the positively charged surface of the photocatalyst particles and the electron pair and carboxylic group of MR at this pH. Therefore, at this pH, the number of hydroxyl groups of the photocatalyst was increased, which facilitates the adsorption of MR.

#### 3.4.5 Effect of scavengers

The main aspects of photocatalysis is making of reactive radicals which used during photodegradation [38]. To assess the mechanism of photocatalytic degradation of GZC over methyl red, the effects of reactive species such as hydroxyl radical (${HO}^{*}$), superoxide radical ($O_{2}^{*}$) and hole ($h^{+}$) on the photo degradation of MR were assessed using constant concentration of dye (0.1 g/L) and photocatalyst (0.2 g/L) at pH = 6.4. In the absence of scavengers % of degradation obtained was 94.8%.

Then it decreased to 72% up on addition of AgNO_3_, although the degradation efficiency become 68.1% and 87.6%, up on the addition of CH_3_OH and NaHCO_3_ were added. The effects indicated that all the scavengers have inhibited the photodegradation effectiveness although the effect of AgNO_3_ and CH_3_OH are more pronounced.

The addition of NaHCO_3_ slightly changed the photodegradation of MR. So the main active species in this reaction is superoxide and hydroxyl radicals. The direct participation of $h^{+}$ appeared to be restricted. Relatively the $h^{+}$ involved ultimately through the reaction of these active species with H_2_O molecules to produce ${HO}^{*}$. Therefor our result indicate that participation of the valence band holes and the conduction band electrons in reduction-oxidation process verifying GZC have highest photodegradation efficiency compared to ZC.

### 3.5 Thermodynamic, kinetic and mechanism of the photocatalyst

**Figure 5:**
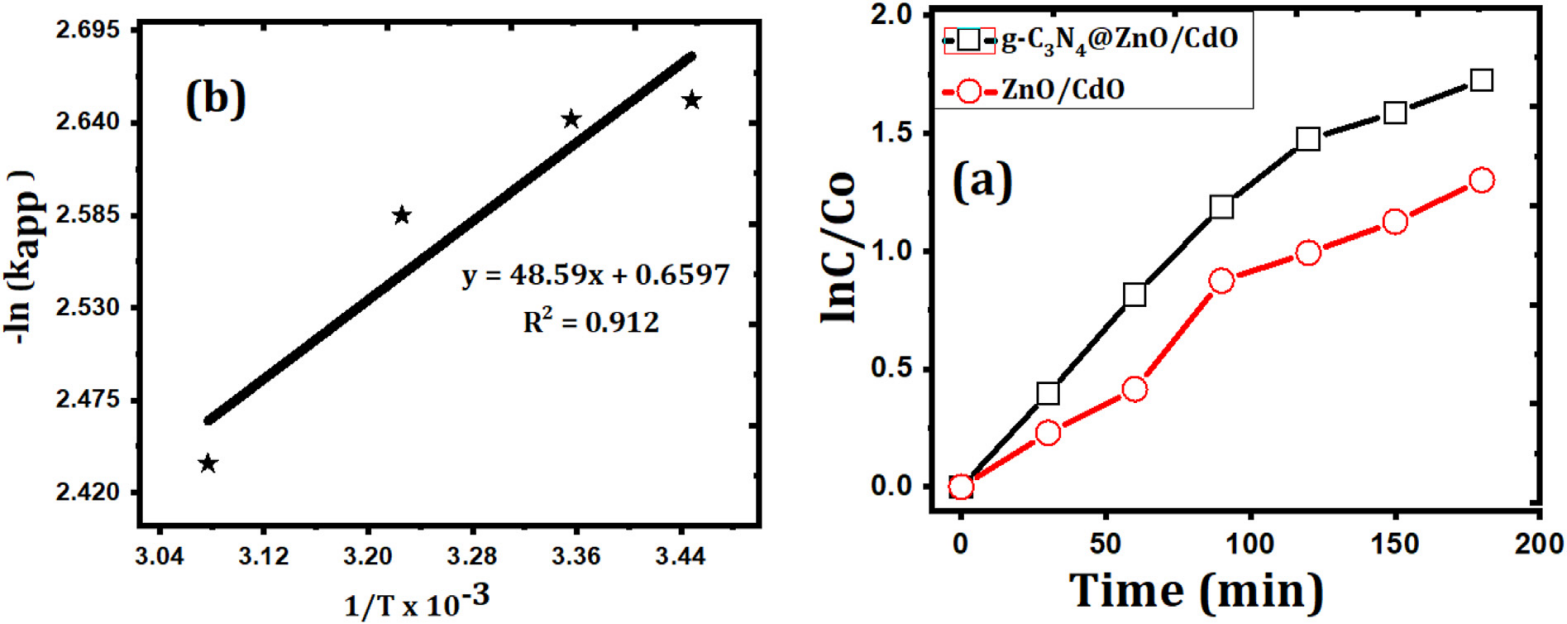
(a) pseudo first-order linear plot of lnC/Co vs time, (b) Arrhenius plot applied for the calculation of activation energy (c, d, e and f) effects of temperature on MR photodegradation (dose of GZC: 0.2 g/L, pH: 6.4, and [MR]: 0.4 g/L).

The kinetics of photodegradation, rate constant (k) and the order of the reactions (n) are fundamental to assess the capability of a photocatalysts. The kinetic investigation of the photodegradation of MR was determined by using different initial concentrations of MR from 0.1 to 1.0 mg/L. Using the graphical technique (Figure 5e), the statistics of ln (C/Co) against time plot is fitted to the linear relationship with a large value of correlation factor (Table 1). It can be suggested that the photodegradation of GZC follows pseudo-first-order reaction.

**Table 1:** Linear correlation and rate constant of the pseudo first-order kinetics for photodegradation of MR.

Photocatalyst	R^2^	k (min^−1^)	$t_{1/2}$
ZnO/CdO	0.986	0.0643	10.8
g-C_3_N_4_@ZnO/CdO	0.997	0.0713	9.72

Meanwhile the temperature at which the reaction proceeds can affect the rate of degradation. Therefore the influence of temperature on photocatalytic degradation process was investigated at different temperatures in the range from 290 K to 325 K. The rate constant and temperature can be described by using Arrhenius equation (Eq. (4)). Plotting of lnKapp versus 1/T gives a straight line (Figure 4e) from which the activation energy can be found and given in Table 2.

(4) lnKapp=lnA−EaRT

where A - the frequency factor, Ea - activation energy of the reaction, Kapp - apparent rate constant, R - ideal gas constant and T-temperature.

**Table 2:** Thermodynamic parameters for the photodegradation of MR by GZC NC.

Parameter	Temperature			
	T = 290 K	T = 298 K	T = 310 K	T = 325
Apparent rate constant ($k_{app}$)	0.0704	0.0712	0.0754	0.0874
Change in Standard free Gibbs energy ($\Delta G^{o}$) in kJ/mol	ΔG=-21.14	$\Delta G^{o}=$-21.58	ΔG=-21.94	ΔG=-22.10
Change in Standard enthalpy ($\Delta H^{o})$	($\Delta H^{o})=$-5.74 kJ/mol			
Change in Standard entropy ($\Delta S^{o})$	($\Delta S^{o})=$ 6.52$\times 10^{-2}$kJ/mol.K			

An increase of photodegradation was detected with rising of temperature (Figure 5c). This is possibly because the collision frequency of molecules in the solution is increased. The values of enthalpy change (ΔH) were calculated using Eq. (5).

(5) $${\Delta H}^{*}=Ea-RT$$

Similarly, to estimate the entropy change of the reaction Eq. (6) is used. Therefore, plotting of ln (k/T) versus ${\Delta H}^{*}/RT$ was done and from its intercept, the value of ΔS∗ was calculated.

(6) lnKappT=ln(KBh)+ΔS∗R−(ΔH∗RT)

Then, the free Gibbs energy was calculated using Eq. (7) [39].

(7) $${\Delta G}^{*}={\Delta H}^{*}-{T\Delta S}^{*}$$

The values of free energy, entropy and enthalpy are given in Table 2. As presented, the MR photodegradation by the GZC attended through a comparatively high negative enthalpy and the Gibbs free energy values, indicating that a spontaneous photodegradation of dye. Also, the weak bonds are exist in the activated complex which allow the situations for the fast degradation of MR.

**Figure 6:**
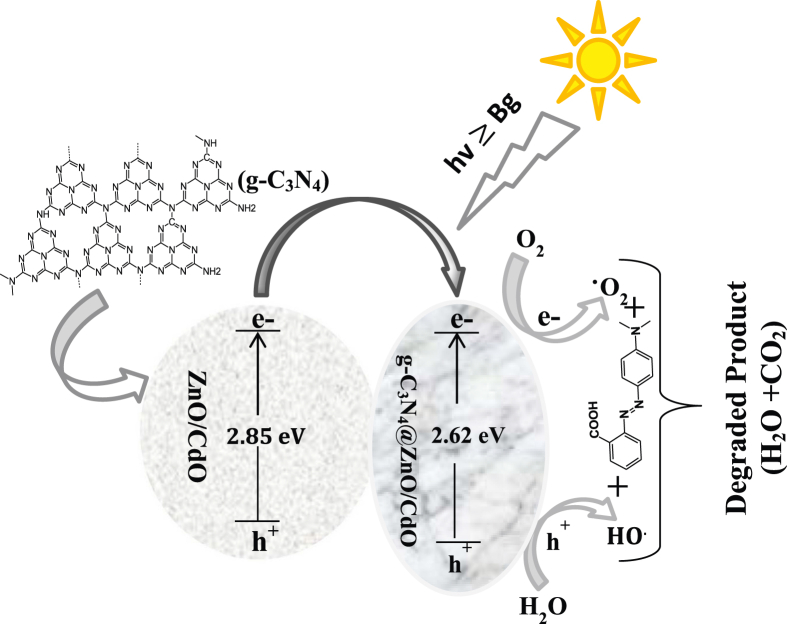
Proposed Mechanism of GZC photocatalytic degradation of MR.

The photocatalytic degradation of MR dye on the surface of the photocatalyst is assumed to happen according to the mechanism revealed in Figure 6. When the photocatalyst was illuminated by UV light the electrons were transfer from the valence bond to conduction bond, h^+^ were created in the VB. This leads to the formation of electron–hole pair.

The $h^{+}$ and $O_{2}^{*}$ combined with H_2_O to form the HO∗ radical. Electrons in the conduction band combined with atmospheric oxygen to form $O_{2}^{*}$ anion. Furthermore, the –OH in the reaction medium is adsorbed in the VB and the ·OH radical additionally facilitated the Photocatalytic activity of MR solutions. Finally, the organic dye solution could be degraded to harmful product. Additionally the degradation efficiency of our nanocomposite was compared with othr literature in Table 3.

**Table 3:** Comparison of ZC and GZC for degradation of MR by various catalysts.

Photocatalyst	Source of Radiation	Degradation efficiency and time	References
ZnO	solar light	86% within 1 h	[40]
CdO	UV light	80.2% within 50 min	[41]
g-C_3_N_4_	Visible light	100% within 30 min	[42]
Ag–N–ZnO	UV light	91.65% within 160 min	[43]
CTAB-TiO_2_	UV light	95% within 2 h	[44]
NiCo_2_O_4_	Visible light	95.1% within 2 h	[45]
TiO_2_/WO_3_	Visible light	95% within 2 h	[46]
TiO_2_–Al_2_O_3_–ZnFeS_2_O_4_	UV light	93% within 1.5 h	[47]
ZnO/CdO	UV light	89.41% within 2 h	This Work
g-C_3_N_4_@ZnO/CdO	UV light	97.78% within 2 h	This Work

### 3.6 Acute toxicity valuation

Doubts related with the potential environmental hazards of nanomaterials are becoming fundamental issue due to their development in different study. Nanomaterials are certainly released in the environment through the fabrication, application and removal process. Wang et al., 2008 reported that ZnO NPs allow the viscosity of blood in mice to be raised, hurt pancreas, spleen, liver as well as other organ [48].

**Figure 7:**
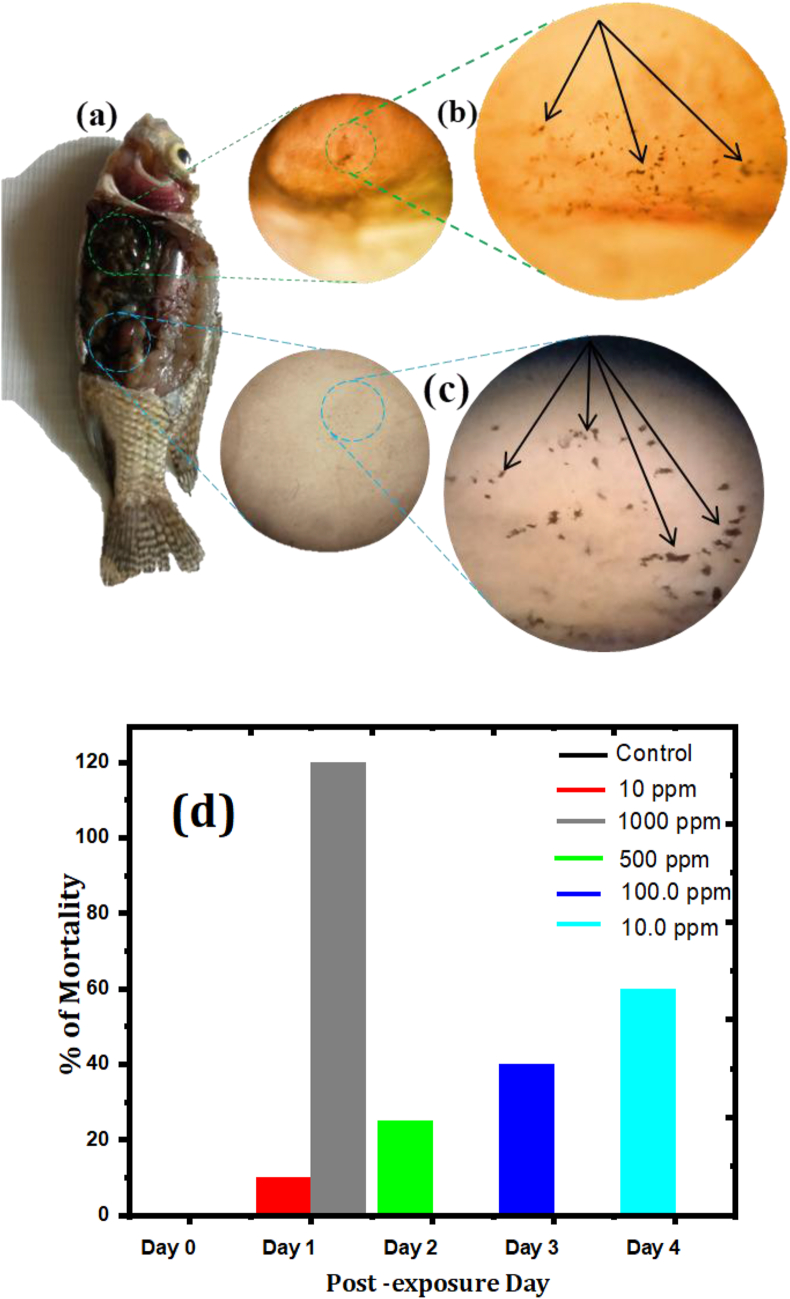
Fish from 1000 ppm g-C_3_N_4_@ZnO/CdO treated group at 12 h exposure displayed patchy black brown emphases dispersed in gill (a), optical microscope images of the ingestion of GZC NCs (black arrows) (×40 magnification) in liver (b), kidney(c) and effects of NCs in terms of % mortality at 12 h. A substantial increase in mortality was seen in 1000 ppm (d).

Also most nanomaterial can cause toxicity injury and may cause damage in DNA of animals after entering to respiratory tract and digestive tract [48, 29, 49]. Figure 7a shows the optical microscope of fish exposed to a solution of 1000 ppm for 24 h. The dark shade seen in the fish shows that these organisms consumed the solution of GZC NCs. It is noted that there are GZC NCs agglomerates saturated in the liver and kidney of the fish (Figure 2b and 2c).

Findings from the experiment show that there were no exposure-related experimental indicators in any lateral periods in the control and 10 ppm GZC treated fish. After a few hours following exposure, Nile tilapia from the 500 and 1000 ppm groups displayed respiratory distress characterized by swimming to the surface of the water, dyspnea and rapid opercula motions. The death rate of tilapias was showed at days 0–4 post-exposure. At day 1 the mortality in the fish treated with 1000 ppm increased noticeably (Figure 7d). The calculated median lethal concentration (LC_50_) for the Nile tilapia at 12 h was 113 ppm [50]. Our findings are well agreement with a previous study, which confirmed that the acute toxicity of Ag nanoparticles to *D. magna* was associated to the accessible concentration of the unconfined Ag ions in the suspension [51]. Donaldson et al. [52] verified that carbon nanotubes could encourage pulmonary irritation over pharyngeal aspiration. Additionally, Hun et al. report for total concentration, the 24 h-EC_50_ values were strongly dependent in the range of 4.2–3844 g L^−1^ for Ag nanoparticle and 54.5–3208 g L^−1^ for CuO nanoparticle [53].

## 4 Conclusion

In summary, ZnO/CdO binary and hybrid g-C_3_N_4_@ZnO/CdO ternary NC were successfully prepared by a precipitation and through facile one-pot ultrasonic assisted techniques. The MR dye degradation efficiency under UV illumination was quantified to measure the photocatalytic performance of the prepared catalyst. Outcomes indicated the highest photodegradation activity attained by GZC NC (97.78%) relative to its ZC (89.41%) counterparts. It has been found that the optimal conditions for photocatalytic degradation of MR were as follows: initial concentration of MR dye is 0.4 g/l, at pH of 6.4, and the concentration of catalyst load is 0.2 g/l under UV light irradiation for 2 h. The effect of scavengers was measured to investigate the mechanism of degradation, superoxide anion radicals and hole were the most reactive component. In the toxicity results of Nile tilapia (*Oreochromis niloticus*), the NCs have toxic efect and the calculated median lethal concentration (LC_50_) was 113 ppm after 12 h.

## Declarations

### Author contribution statement

Sintayehu Berhanu; Haftom Gebremariam: Performed the experiments; Analyzed and interpreted the data; Contributed reagents, materials, analysis tools or data; Wrote the paper.

Samuel Chufamo: Analyzed and interpreted the data; Wrote the paper.

### Funding statement

This research did not receive any specific grant from funding agencies in the public, commercial, or not-for-profit sectors.

### Data availability statement

Data included in article/supp. material/referenced in article.

### Declaration of interest’s statement

The authors declare no conflict of interest.

### Additional information

No additional information is available for this paper.
